# Lithium-ion battery second life: pathways, challenges and outlook

**DOI:** 10.3389/fchem.2024.1358417

**Published:** 2024-04-08

**Authors:** Anisha N. Patel, Laura Lander, Jyoti Ahuja, James Bulman, James K. H. Lum, Julian O. D. Pople, Alastair Hales, Yatish Patel, Jacqueline S. Edge

**Affiliations:** ^1^ Department of Mechanical Engineering, Imperial College London, London, United Kingdom; ^2^ Department of Engineering, King’s College London, London, United Kingdom; ^3^ Birmingham Law School, University of Birmingham, Birmingham, United Kingdom; ^4^ Department of Mechanical Engineering, University of Bristol, Bristol, United Kingdom; ^5^ Department of Engineering, University of Exeter, Exeter, United Kingdom; ^6^ The Faraday Institution, Didcot, United Kingdom

**Keywords:** lithium-ion battery, end-of-life, second life, repurposing, state-of-health, safety, policy, regulation

## Abstract

Net zero targets have resulted in a drive to decarbonise the transport sector worldwide through electrification. This has, in turn, led to an exponentially growing battery market and, conversely, increasing attention on how we can reduce the environmental impact of batteries and promote a more efficient circular economy to achieve real net zero. As these batteries reach the end of their first life, challenges arise as to how to collect and process them, in order to maximise their economical use before finally being recycled. Despite the growing body of work around this topic, the decision-making process on which pathways batteries could take is not yet well understood, and clear policies and standards to support implementation of processes and infrastructure are still lacking. Requirements and challenges behind recycling and second life applications are complex and continue being defined in industry and academia. Both pathways rely on cell collection, selection and processing, and are confronted with the complexities of pack disassembly, as well as a diversity of cell chemistries, state-of-health, size, and form factor. There are several opportunities to address these barriers, such as standardisation of battery design and reviewing the criteria for a battery’s end-of-life. These revisions could potentially improve the overall sustainability of batteries, but may require policies to drive such transformation across the industry. The influence of policies in triggering a pattern of behaviour that favours one pathway over another are examined and suggestions are made for policy amendments that could support a second life pipeline, while encouraging the development of an efficient recycling industry. This review explains the different pathways that end-of-life EV batteries could follow, either immediate recycling or service in one of a variety of second life applications, before eventual recycling. The challenges and barriers to each pathway are discussed, taking into account their relative environmental and economic feasibility and competing advantages and disadvantages of each. The review identifies key areas where processes need to be simplified and decision criteria clearly defined, so that optimal pathways can be rapidly determined for each end-of-life battery.

## 1 Introduction: Pathways for end-of-life batteries

The race towards global electrification and zero carbon emission is raising new challenges, notably the surge in end-of-life (EoL) lithium-ion batteries (LiBs) from electric vehicles (EVs). By 2025, it is estimated that over 800,000 metric tons of EV batteries worldwide (Wu et al., 2020). EoL is defined as when a battery reaches 70%–80% of its original storage capacity (Wood et al., 2011; Lih et al., 2012; Lacey et al., 2013; Ambrose et al., 2014; Martinez-Laserna et al., 2018). This 80% EoL criterion was established for nickel-cadmium batteries (Martinez-Laserna et al., 2018). Despite the uptake of modern LiBs, the 80% criterion is still in use to define the EoL of all EV packs, including those using lithium technologies. LiBs have far greater energy [240–300 Wh/kg (Global, 2020)], power [200–950 W/kg (Dechent et al., 2021)] and longer lifetimes [6–15 years (Ambrose et al., 2014)] than their traditional counterparts. It is estimated that by 2030 there will be up to 120 GWh/year of wasted, untapped resource stored in EoL batteries (Global, 2020). This is driving a new field of research into ways of *reusing* EoL batteries in further, less demanding, automotive applications (Ramoni and Zhang, 2013; Martinez-Laserna et al., 2018). Furthermore, different applications can tolerate lower state-of-health (SoH) values without significant compromises on performance or safety, hence the EoL criteria is an overly conservative definition that can be greatly improved by considering chemistry and application.

In this review, we highlight the issues with disposal before focussing on recycling and repurposing pathways, holistically bringing together technical, economic, policy and environmental perspectives for the first time. We outline current strategies for deciding EoL battery pathways, discussing key challenges, as well as technical barriers, that must be overcome.

**FIGURE 1:**
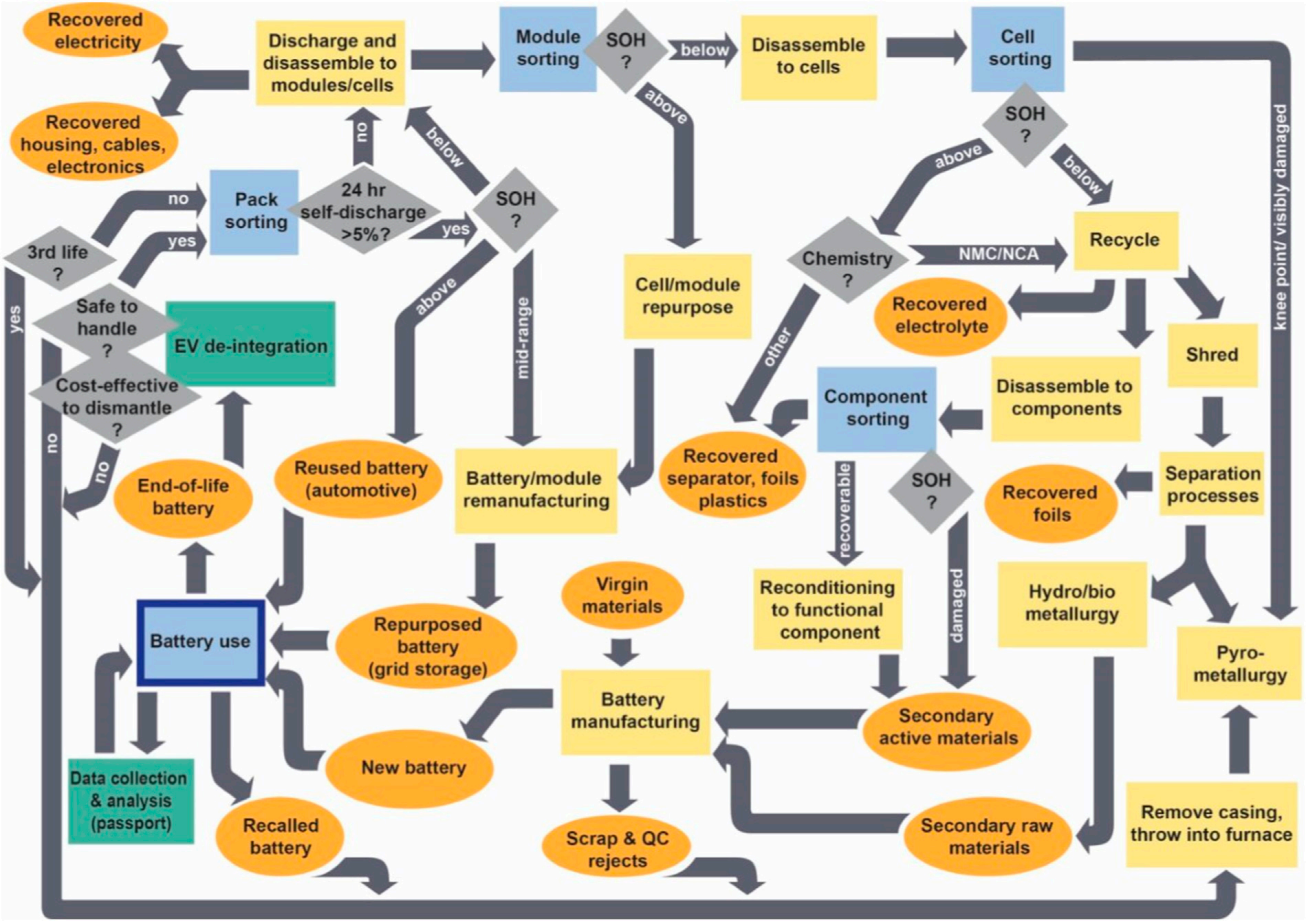
A flowchart showing the end-of-life (EoL) pathways for the battery lifecycle, including decisions which need to be made at specific stages. Qualitative ranges have been selected, as the actual figures may change over time. For example, at present, when deciding which route to choose for an EoL battery pack, based on the state-of-health (SoH) check, those which exceed 80% (“above”) are suitable for reuse in another automotive application; those which demonstrate 50% or less (“below”) must be dismantled and those which lie between these limits (“mid-range”) can be considered for repurposing. Reprinted with permission from Edge, J. S., DOI:10.5281/zenodo.10257443 Preprint at 2023.

Once a battery has reached the EoL for its primary use, it can follow one of four pathways, as described in Figure 1 and summarised as follows (Engel et al., 2019):

(i) direct disposal, in which batteries are placed in landfills;
(ii) recycling, for resource recovery;
(iii) *reuse* in an alternative automotive application, followed by recycling or disposal;
(iv) *repurposing* for a second life application, followed by recycling or disposal.

The first option presents an environmental hazard (Mrozik et al., 2021), while the remaining three options rely on battery collection and sorting, providing additional logistical complexity and costs to the battery life cycle. Since batteries are designed and manufactured for the requirements of their first life application, they are not necessarily optimised for use in other applications. Reuse or repurposing may therefore require additional processes to restore the electrochemical function of the recovered materials, cells or packs and make them suitable for further use. Regardless of whether batteries are reused or repurposed, they will eventually become unusable and then require recycling, to recover the valuable materials within and avoid the hazards of a build-up of spent batteries or their disposal into landfills. The additional costs of treating EoL batteries can, in some cases, be offset by the continued services or reusable materials extracted from them (Standridge et al., 2014), but the global processes for LiB reuse, repurposing and recycling have yet to achieve maturity.

Direct disposal of LiBs can introduce environmental and health hazards, due to the toxic materials they contain, such as cobalt, lithium and nickel, which can leach into soil and water or be released into the air, posing long-term detrimental effects to human and ecosystem health. Hence, strict regulations exist globally on the handling and treatment of waste LiBs (Leba et al., 2018; Mrozik et al., 2021). While many countries may introduce similar restrictions, it is conceivable that there will be countries which are less well regulated.

LiBs are a complex mix of reactive materials and will degrade over time, even if unused. EoL batteries are likely to be degraded, potentially damaged and will become severely undercharged over time, possibly undergoing a thermal runaway event and igniting a landfill fire, which would be very difficult to suppress and cause considerable pollution. LiB fires are themselves especially hazardous, difficult to extinguish and produce many toxic and environmentally harmful vapours (Bravo Diaz et al., 2020). The scale of future EV battery waste makes it imperative to find solutions to safely collect and, at the very least, dismantle LiBs. One alternative solution proposed is to stockpile battery packs until the economics of further processing become favourable (Pool, 2020). However, the risks of thermal runaway remain and there have been a number of cases of fires breaking out, even where the stockpiled batteries were newly produced (Sanderson, 2021). Enhanced temperature control in storage may mitigate these risks, but this would add significantly to cost and environmental impact.

One alternative to disposal is recycling. Many of the materials within a LiB are listed as critical, as they are not widely available as natural resources (Olivetti et al., 2017). Recycling of LiBs is an important way to recover the valuable materials within, reducing the demand for virgin materials and the environmental impacts associated with extracting them. Recycling also reduces the risks of degraded LiBs starting fires, wherever they are disposed of. While there are options for reusing batteries in second life applications, there will ultimately be the need to recycle them.

There are four main recycling methods that are actively being researched or in use in industry: (i) pyrometallurgy, (ii) hydrometallurgy, (iii) biometallurgy and (iv) direct recycling. The advantages and disadvantages of each are summarised in Table 1. The pyrometallurgical approach is versatile and fairly straightforward to deploy and is therefore the main method currently deployed in industry. However, it is labour-intensive and can only extract a limited set of metals (nickel, aluminium, cobalt, copper) via chemical reactions occurring at very high temperatures (Dunn et al., 2012a; Gaines et al., 2018a; Chen et al., 2019a; Liu et al., 2019). This can lead to large quantities of CO_2_ being emitted and, for some LiB cathode chemistries, can even result in a net increase of carbon emissions, as is the case for lithium iron phosphate (LFP) (Ciez and Whitacre, 2019). Hydrometallurgy uses a sequence of solvent-based chemical steps (Chen et al., 2019a) and operates at much lower temperatures than pyrometallurgy, thus having a lower energy consumption. However, the pre-treatment steps of disassembly, separation and grinding are essential. This method can extract a wider range of the component materials, including cathode metals, lithium and graphite and can be used in conjunction with pyrometallurgy. An emerging alternative to pyro- and hydrometallurgy is biometallurgy, including biomineralisation (Patel et al., 2021) and biological leaching or bioleaching (Ferrara et al., 2021; Biswal and Balasubramanian, 2023), which has been used successfully in the mining industry. This technique uses bacterial or fungal microbes to either reduce metal oxides into harvestable nanoparticles or to produce the acids required to leach out metals and is of particular interest for the separation of metals such as nickel and cobalt, which are inherently difficult to separate and currently require either sequential precipitation or extraction with organic solvents (Harper et al., 2019a).

**TABLE 1:** Advantages and disadvantages of recycling methodologies, including those currently available and under development. Table data summarised from Ganter et al. (2014), Gaines et al. (2018b), Chen et al. (2019a), Lander et al. (2021), with specific references noted where appropriate.

Recycling method	Advantages	Disadvantages
Pyrometallurgical recycling	Suitable for any battery composition/chemistry	Energy-intensive
	No pre-treatment (separation, discharge, crushing, etc.) required	Additional processing required to extract metals from slag (particularly lithium)
	High recovery of metals	Economic viability depends on battery compositions containing certain valuable metals, e.g., cobalt Gaines et al. (2018b), Dunn et al. (2021), Lander et al. (2021)
	Process path is simple, easy to scale up	Cannot be used for lithium iron phosphate composition
	No safety hazards regarding leakage of chemicals from battery	
	In commercial use	
Hydrometallurgical recycling	Suitable for any battery composition/chemistry	Cells must be discharged, sorted and crushed or shredded, increasing complexity, length and cost of process
	Energy efficient relative to pyrometallurgy, due to low temperatures used	High levels of waste (effluent) which needs disposal/recycling
	High purity of extracted metals	Use of solvents, although some may be recoverable for reuse
	Can be the cheapest method Lander et al. (2021)	Water consumption
Biometallurgical recycling	Can be tailored for many battery compositions/chemistries	Cells must be discharged, sorted and crushed or shredded, increasing complexity, length and cost of process
	Energy efficient relative to pyrometallurgy, due to low temperatures used	Biological culturing times are slow and require carefully controlled conditions
	High purity of extracted metals	
Direct recycling	Able to recover a wide range of component materials	Cells must be discharged, sorted and dismantled, increasing complexity, length and cost of process. This could be helped with mandatory labelling of cells, giving their material composition Thompson et al. (2020)
	Active materials can be reused straight after recovery, although some reconditioning may be required	Materials recovered from the process may not function to the same capability as new material, due to structural changes occurring through battery ageing. Restoration processes may be required Ganter et al. (2014)
	May be the only option for battery chemistries having low value materials, such as lithium iron phosphate or sodium-ion batteries Lander et al. (2021)	Each chemical composition will require its own recovery process. One exact method cannot be applied to every battery type
		Inflexible process recovering electrode materials in their composite form, but those materials may cease to be useful in that form, due to advances in battery technology
		Requires commercial scale-up: currently only at lab level

By combining all the component materials, these metallurgical approaches need additional processes to separate out the pure metals, which will then be recombined through further processing to make a new battery (Dunn et al., 2012a). Direct recycling uses a different approach, employing mechanical and solvent-based separation methods to recover composite battery materials, such as active cathode, for direct reuse in new batteries, requiring minimal further processing (Dunn et al., 2012a). Overall, direct recycling processes are able to recover a greater range of component materials, including non-metallics, while producing the lowest greenhouse gas emissions. However, they are still primarily at the laboratory scale and therefore merit further research and investment to progress the methods to commercial scales (Dechent et al., 2021; Edge et al., 2021).

Recycling LiBs presents several challenges that need to be addressed to ensure effective and sustainable recycling processes. As the recycling processes themselves involve energy consumption, emissions and waste generation, the environmental impacts of recycling may sometimes outweigh the benefits. Improving the efficiency and yield of resource recovery processes will be crucial to maximize the value and sustainability of recycling.

Recycling methods rely on a range of pretreatment processes for spent batteries, such as collection, dismantling, separation and sorting. Batteries are dispersed across various consumer products and industries, making it challenging to collect them efficiently and the lack of proper labelling makes it difficult to choose the most appropriate recycling pathway (Zhao et al., 2021a). Additionally, batteries come in different shapes, sizes, and chemistries, requiring specialized and highly complex sorting systems to separate them effectively (Beaudet et al., 2020). In order to improve recycling yield, it is necessary to dismantle battery packs, modules and cells. This is a labour intensive, costly process and recommendations have been made for the design of battery modules and packs to enable easier dismantling, so that recycling processes can be streamlined (Gaines et al., 2018a; Thompson et al., 2020). Combined with the need for specialized equipment, facilities, and skilled labour, the cost of recycling and reusing materials to make raw batteries is currently higher than the cost of mining raw materials (Beaudet et al., 2020). For example, the current cost of recycling lithium has been estimated to be three times the cost of mining lithium (Melin, 2019a).

Current recycling pathways mainly target the more valuable base metals such as cobalt, manganese, nickel and copper, relative to the less valuable lithium. Vital resources critical to LiB manufacturing, such as cobalt, are becoming prohibitively expensive, and impacted by geopolitical challenges (Harper et al., 2019a; Li et al., 2020). However, one major challenge facing recycling is the need to adapt to the fast-evolving battery chemistries, particularly as EV batteries have long lives of many years and sometimes decades. As cheaper battery chemistries are developed, recycled materials become less profitable and may no longer be required for the production of the latest chemistries. Governmental policies are needed to drive developments in the recycling industry, so that these potentially incendiary devices do not end up in landfills.

By extending the lifespan of lithium-ion batteries through reuse and repurposing, the immediate need for recycling is reduced, lessening the environmental impact associated with recycling processes and reducing the risk of large-scale LiB disposal because no viable alternative pathway exists. Second life batteries (SLBs), also referred to as retired or repurposed batteries, are lithium-ion batteries that have reached the end of their primary use in applications such as electric vehicles and renewable energy systems (Zhu et al., 2021a). Rather than being discarded or immediately recycled, these batteries are repurposed in new applications. Despite no longer meeting the requirements of their original intended use (usually in automotive), SLBs retain a significant portion of their capacity and functionality. These batteries have many viable applications in a second life format; for example, to provide an energy store within our grid energy networks, to complement the intermittent loading associated with renewable energy harvesting methods (Zhu et al., 2021a; Martinez-Laserna et al., 2018).

However, material flow analysis highlights that there are trade-offs between the environmental benefits, economic values, and resource optimisation for SLBs. The second life pathway delays the recirculation of valuable materials, whose supply chains can become more vulnerable to disruption given their existing supply risks, compared to the case of direct recycling after their first life (Tao et al., 2021a).

Battery reuse is becoming a global priority given the environmental impact and impetus to extract maximum value from the valuable materials used in battery manufacture: reuse generally ranks high as a sustainable end-of-life pathway (Zhao et al., 2021a). There are, at present, no universal or global standards or regulations for managing end-of-life LiBs. Nations across the world vary widely in their approach to balancing economic and environmental objectives; as well as availability of technological and recycling infrastructure. Thus, different countries frame their own unique regulations and strategies for managing LiB at the end of first life. For example, China has relatively strict regulations around recycling and decarbonising the supply chain; while in the US, in contrast, legislation to promote a circular economy in EV LiBs is lagging behind both China and the EU (Global, 2021).

## 2 Second life: opportunities and pathways

EV batteries are both environmentally and economically expensive to manufacture, therefore extending their service life can offset these costs (Foster et al., 2014; Harper et al., 2019a). Although EoL batteries are no longer suitable for the power and energy demands of most automotive applications, they can still be useful for grid-storage applications, which are often not restricted by weight nor volume. Repurposing of SLBs has the potential to be less expensive than deploying new batteries of equivalent performance and increases the products’ useful life, enhancing the lifetime energy throughput as a ratio of the energy spent on manufacturing, therefore offering an environmentally sustainable option supporting a circular battery economy (Jiao and Evans, 2016a; Harper et al., 2019a). By understanding the legal standards, pathways for repurposing, and steps to meeting those standards, and addressing these aspects, we can unlock the full potential of SLBs.

### 2.1 Defining end-of-life and challenges for second life

The US Advanced Battery Consortium defines EV battery EoL as the point at which the battery reaches 80% of its original rated capacity or 80% of its original power capability at 80% Depth of Discharge (Hunt, 1996). The rationale for this is a concern that degradation beyond this may result in the battery not being able to serve surge current drawn during acceleration. However, the 80% threshold was established in the 1990s when nickel-based batteries, having lower energy and power densities than LiBs, were used in most EVs. Recent studies have shown that lithium-ion EV batteries with 80% remaining capacity can still meet the daily travel needs of over 65% of US drivers (Saxena et al., 2015), indicating that the current EoL criteria may not be suitable, and that the industry should evolve to adopt EoL criteria that match the performance characteristics of LiBs.

Defining battery EoL is challenging, but battery health assessment and clear EoL criteria are critical for safe operation of EVs (Wenzl et al., 2005). One EoL approach proposes a multi-dimensional EoL threshold, based on the match between the battery and application characteristics, i.e., on how the EV is being used and the battery’s suitability for this particular application (Arrinda et al., 2021). However, evaluation of the EV battery’s expected performance is complicated by the difficulty of conducting tests *ex situ*. Ideally, the EoL criteria used for EV batteries must be measurable, either through the onboard battery management system (BMS), or through use of very simple, non-invasive, external hardware and software. Whilst beyond the scope of this review, battery SoH evaluation is an important requirement for extending and optimising the first life of EV batteries.

While automotive LiBs may have exhausted their life within an EV and no longer meet the power demands for the primary use, these LiBs may still be viable for lower power applications, such as electric golf carts, scooters, and some industrial/commercial vehicles. While this is still a “second life” application, the literature tends to refer to second life as repurposing the battery for a different, non-automotive application.

### 2.2 Repurposing applications

With the future annual supply of SLBs predicted to be 112–227 GWh in 2030 and the global grid storage demand estimated at 183 GWh (Engel et al., 2019), SLBs present a significant economic and environmental opportunity to meet all our grid storage needs (Engel et al., 2019), displacing the impacts of manufacturing new batteries for these applications.

The rationale for deploying “retired” EV battery packs in grid storage applications is to extend the service life of the battery, thereby reducing costs and carbon emissions (Martinez-Laserna et al., 2018), when considering these over the whole battery’s lifetime ($/equivalent full cycle and kg CO_2_/equivalent full cycle) (Martinez-Laserna et al., 2018). Additionally, extending the battery’s useful life will delay the immediate need for recycling, allowing the battery recycling industry time to develop. This is expected to reduce the likelihood of illegal battery disposal. However, it will also significantly delay access to the materials, many of which are valuable and critical and therefore needed to both boost the recycling industry and displace the impacts of extracting raw materials. The conflict between these two options is still being debated (Gaines, 2018; Harper et al., 2019a; Dunn et al., 2021).

While some retired EV battery packs retain only a small amount of their original capacity or power capability, they can still be viable for low-demand applications, which require only low current rates and shallow depth of discharges. However, different grid storage applications have vastly different power requirements, for example, grid frequency modulation is significantly less demanding than any peak shaving application. Therefore, a sensible beginning-of-second-life health check can ensure repurposed batteries are used in suitable applications (Martinez-Laserna et al., 2018).

The applications most suitable are those that require less frequent battery cycling (Engel et al., 2019). The potential applications of repurposed batteries span scales from residential storage through to large-scale grid support. Table 2 lists the capacity ranges required for each potential application, indicating whether repurposed batteries are suitable. Residential, commercial, and industrial applications of repurposed batteries collectively contribute to grid stability by regulating frequency and distributing demand across extended time frames. Utility scale operations require very large amounts of energy to be stored and released and are less suited to repurposed batteries, due to their reduced capacities and ongoing degradation, as well as safety concerns when operating at very high power requirements, at C-rates (a measure of the current at which the battery is charged or discharged) exceeding 2C (Mcloughlin and Conlon, 2015).

**TABLE 2:** Viability of each application in each sector with their capacity ranges. Green indicates that repurposed batteries are suitable for this application; orange indicates that they are only sometimes suitable and red indicates that they are not suitable. Capacities in residential applications (e.g., homes and apartments) typically range from 3 to 20 kWh, depending on factors such as number of occupants and building size. Capacities within the commercial sector (buildings or spaces specifically allocated for business activities) range from 3 to 500 kWh, contingent upon the scale and energy demands of the business. Capacities in the industrial sector (areas designated for the manufacturing and processing of goods and services) span from 500 kWh to several megawatt-hours. Green cells indicate application areas which are deemed to be viable for SLBs, supported by academic literature and insights gained from prior pilot programs focused on SLBs. The orange ranges indicate that a paper suggested or briefly indicated the potential viability of an SLB for a given application. Red ranges indicate applications which are deemed to be unsuitable for SLBs, either due to unfavourable economic feasibility or the need for excessively high C-rates. Relevant sources have been cited in place, but most ranges are summarised from the installations shown in Table 3.

	Residential	Commercial	Industrial
**Renewables integration**	0-150 kWh	0-500 kWh	0-10 MWh Mcloughlin and Conlon (2015)
**Energy arbitrage**	0-60 kWh	0-500 kWh	N/A
**Peak load shaving**	0-60 kWh	500-4,500 kWh Mcloughlin and Conlon (2015)	0-4 MWh Mcloughlin and Conlon (2015)
**Back-up power**	0-40 kWh FAQ (2022)	0-700 kWh Mcloughlin and Conlon (2015)	0-4 MWh
**Small mobility vehicles**	0-15.3 kWh Montes et al. (2022a); Lamedica et al. (2022)	0-8 kWh Montes et al. (2022a), Phophongviwat et al. (2023)	11-25 kWh Jiao et al. (2021), Montes et al. (2022a)
**EV charging**	0-20 kWh transportation (2022)	0-1 MWh transportation (2022)	0-5 MWh transportation (2022)
**Demand response**	N/A	0-2 MWh	0-4 MWh
**Microgrid**	0.02-2 MWh Microgrids (2022)	2-6 MWh	6-20 MWh

Peak load shaving is the grid-level practice of storing energy when it is abundant and demand is low, then releasing it during low energy availability and high demand. This is beneficial for reducing peak load, buffering the variability of grid energy demand, and smoothing the intermittency of renewables, such as solar and wind energy. Currently, peak demand is managed with expensive, polluting reserve generators, using energy sources such as natural gas or diesel (Hanley et al., 2009). Repurposed batteries could replace these, but only for smaller scale applications.

Microgrids are localised electrical grids of growing importance, somewhat isolated from the wider national grid, but are expected to be integrated into the wider grid infrastructure in the future (Hanley et al., 2009). Battery energy storage systems (BESS) encourage the development of microgrids for rural villages which are scattered across vast areas of land and can be decoupled from a centralised grid. Currently, remote areas receive power via diesel generators; repurposed batteries could provide a cost-effective, low carbon alternative for providing flexibility and reliability, maintaining power quality and boosting supply during peak demand.

Residents in urban areas with uncertain grid infrastructure require a reliable back-up power source, particularly to preserve food supplies and enable internet access during instances of grid unreliability. Residential back-up and uninterruptible power supply applications are suitable for repurposed battery systems. Industrial applications benefiting from this capability include data centres, medical establishments, airports, and emergency response hubs.

Charging an EV at a residential house requires a substantial power output, typically ranging between 20 and 60 kWh, depending on the vehicle. Company and industrial EV charging requirements have surged as fleets undertake the transition to electrification. Consequently, this and the integration of rapid charging stations creates high costs and energy demands on the grid. Repurposed batteries have proven to be a viable solution to supply surplus electricity to manage charging loads, ensuring a consistent and dependable charging experience (Montes et al., 2022a). This contributes holistically to the wider adoption of EVs, as it optimises the charging infrastructure.

Pilot projects are a useful way to demonstrate the capabilities of used EV batteries and determine installation and operational logistics. To illustrate the range of second-life applications being considered, a select list of pilot projects is given in Table 3.

**TABLE 3:** Pilot and commercial projects since 2012, which use second life lithium-ion batteries [updated from Reinhardt et al. (2019) and Zhu et al. (2021a)]. The information is based on press releases and may not be complete or reflect the final characteristics of installations.

OEM	Partners/Service provider	Cells	Power/Capacity	Year	Application	Country
Ford	Duke energy, ITOCHU	TH!nk	2 MWh	2010	Renewable power, Fast charging	Japan, United States (Indianapolis)
GM	ABB	Chevrolet Volt	25 kW/50 kWh	2012	Power supply, renewable energy storage	United States
Nissan	Sumitomo, 4R Energy	Nissan Leaf	600 kW/400 kWh	2013	Island power, Grid services, Renewable energy storage	Japan
Nissan	Eaton, CEA, EPFL, ICTroom, Credit Suisse, University of Trento			2014	UPS, data centre	
BMW	UC San Diego	Mini-E	108 kW/180 kWh	2014–2017	Fast charging	United States (San Diego)
Nissan	Relectrify	Nissan Leaf	60 kWh	2015	Grid services and renewable energy	United States, Australia
GM			17.1 kWh/pack	2015	Data centre, Renewable energy storage	
Toyota	2nd life battery LLC	208 Camry modules	85 kWh	2015	Renewable energy	United States (Yellowstone National Park)
BMW	Beck Automation	i3	22 or 33 kWh/pack	2016	Residential energy storage	Canada
BMW	Vattenfall and Bosch	ActiveE and i3	2 MW/2.8 MWh	2016	Renewable energy storage	Germany (Hamburg)
Mercedes-Benz	GETEC Energie, The Mobility House, Remondis	Smart fortwo	12 MW/13 MWh	2016	Recycling plant	Germany (Lünen)
Nissan	Amsterdam Arena	280 Nissan Leaf	5.6 MWh	2016	Back-up power	Netherlands (Amsterdam)
Renault	Powervault	JXTG		2017	Renewable energy storage and grid stability	Japan
Renault	United Technologies Research Centre Ireland, Ltd	Renault Kangoo	88 kWh	2017	Renewable energy, Peak load shaving	France, Italy, United Kingdom, Germany
Renault	City of Terni, ASM Terni	Renault Kangoo	66 kWh	2017	Peak shaving, Power quality, Grid Services, Renewable energy	Italy (Terni)
Nissan	Eaton	Nissan Leaf	4.2 kWh	2017	Residential energy storage	United Kingdom
Nissan	Gateshead College, United Technologies Research Centre Ireland, Ltd	Nissan Leaf	48 kWh (50 kWh PV capacity)	2017	Research, renewable energy storage	United Kingdom (Sunderland)
Nissan		12 Nissan Leaf	192 kWh	2017	EV charging and renewable energy	France (Paris)
Nissan	Eaton, The Mobility House	Nissan Leaf	4 MW/4 MWh	2018	Peak shaving, back-up power	Netherlands
BMW	Vattenfall	i3	50 kW/12 kWh	2018	Fast charging	Germany
BMW	EVgo	2 i3	30 kW/44 kWh	2018	EV charging	United States (Los Angeles)
Renault	Nidec, The Mobility House		50 MWh	2018	Grid storage	France
Renault	Connected Energy	Renault Zoe	360 kWh	2018	Fast charging	United Kingdom
Renault	Morbihan Energies, Les Cars Bleus and Enedis			2018	Resort power	France (Belle-ile-en-Mer)
Renault	Sustainable Porto Santo—Smart Fossil Free Island (Empresa de Electricidade da Madeira, The Mobility House, Bouygues Energies et Services, ABB)	Renault Zoe and Kangoo Z.E.		2018	Island power, smart charging	Portugal (Porto Santo)
Audi	Belectric		1.9 MW/22 MWh	2018	Renewable energy, Grid services (frequency response)	United Kingdom, Germany
Toyota	Chubu Electric power, Tokyo Electric Power Company		10 MW	2018	Power capacity, Power quality	Japan
Toyota	JERA		485 kW/1,260 kWh	2018	Power capacity, Power quality	Japan
Nissan	The Mobility House, Empresa de Electricidade da Madeira	Nissan Leaf	N/A	2018	Island power	Portugal
Nissan	Sumitomo, 4R Energy			2018	Rebuilt replacement LiBs	
Toyota	Seven Eleven		10 kWh/unit	2018	Back-up power	
Nissan	EDF	Nissan Leaf		2018	Demand-side platform, powershift	United Kingdom
Volvo Group	Goteborg Energi, Riksbyggen, Johannesburg, Science Park	Electric truck and bus batteries	200 kWh	2018	Residential SLB, Renewable energy	Germany, South Africa
Nissan			700 Wh/device	2019	Portable ES, camping trailers	
Nissan	Eaton, BAM, The Mobility House	148 Nissan Leaf (42% 2nd life)	3 MW/2.8 MWh	2019	Grid services	Netherlands, (Amsterdam)
Daimler	BAIC	BJEV	40 MWh	2019	Second life parts storage unit sector	China
BYD	Itochu		1 MWh	2019	Grid storage	Australia and Southeast Asia
	BAK, China Southern Grid		0.15 MW/7.27 MWh	2019	Grid storage	China
Renault	ENGIE, Umicore	48 Renault Kangoo	1.2 MW/720 kWh	2019	Grid storage	Belgium
Audi	EnBW	e-tron		2020	Grid storage	Germany (Helibronn)
GM	SAIC, Wulin	Baojun E100 and E200	250 kW/1 MWh	2020	Grid storage	China
Renault	SmartHubs (Connected Energy, Moixa, PassivSystems and ICAX, Newcastle University, West Sussex County Council and Innovate United Kingdom)	Renault Kangoo, mixed old and new cells	50 kW/14.5 MWh 70 MW/60 MWh	2020	Residential and business energy storage, four sites planned	United Kingdom (West Sussex)
Renault	Advanced Battery Storage (Nidec, The Mobility House, Demeter, Banque des Territoires)	Mixed old and new cells	50 MWh	2020	Residential and business energy storage, Several sites planned	France (Douai)
Renault	Connected Energy	Renault Kangoo	360 kWh, 720 kWh, 1.2 MWh	2020	Fast charging, renewable energy, Grid storage	Belgium
Renault	City of Kempten, the Allgäuer Überlandwerk GmbH	6 Renault Kangoo	95 kWh	2020	Micro-grid, renewable energy	Germany (Kempten)
Toyota	Eurus energy, Tokyo Electric Power Co. Holdings		1 MW/3 MWh	2020	Grid storage	Japan
Volvo Group	Batteryloop	Electric truck and bus batteries	200 kWh	2020	Renewable energy, Microgrid	Sweden
Audi	RWE	60 packs	4.5 MWh	2021	Renewable energy	Germany (Berlin)
Renault	Eco2Charge	Kangoo ZE	66 kWh	2021	Renewable energy	France
Mitsubishi and PSA	EDF and Forsee Power	Peugeot Ion, C-zero, iMiev		2022	Renewable energy	France
Mercedes-Benz	Moment energy		60 kWh	2022	Microgrid, Renewable energy	Canada
Honda	B2U	Clarity	3 MW/12 MWh	2023	Renewable energy	United States (California)
Mercedes	Batteryloop		2.8 MW/2.6 MWh	2023	Residential power	Sweden (Gothenburg)
Mixed	CleanMobilEnergy (Connected Energy, Engie, Innovate United Kingdom, EU)	24 EV batteries	600 kW/600 kWh	2023	V2G	United Kingdom (Nottingham)
Mercedes-Benz	Envon		26 MWh	2023		Norway
Nissan, Tesla, Ford, Chevy	B2U	1,300 packs	28 MWh	2023	Grid services	United States (California)
Renault	Swarco Smart Charging, Connected Energy	24 Renault Kangoos	300 kW	2023	EV charging	United Kingdom
Audi	Mobility House	20 e-tron	1.25 MW/1.9 MWh	2023	EV charging, Grid services	Germany (Berlin)
BMW	UC San Diego	Mini-E	100 kW/60 kWh	2023	Renewable energy	United States (San Diego)
BMW	Tricera	Rolls Royce	100 kWh/300 kWh/2 MWh	2023	Peak load shaving, EV charging, renewable energy	Germany
Forsee	Connected Energy	Forsee lithium nickel manganese cobalt oxide (Zen 4 and Zen 35)	40 MWh	2024	Grid storage	United Kingdom
Volvo Group	Connected Energy	Electric truck and bus batteries	1 MWh	2024	Grid storage, Ports	United Kingdom

### 2.3 Requirements for second life

Retired EV batteries must undergo a series of tests to ensure that they are suitable for second life applications. This section discusses the relevant safety standards and SoH assessment which are essential for determining suitability for repurposing. In some cases, whole packs may be repurposed in a second life application, but this application may have new requirements requiring the pack to be reconfigured. In this case, there may be further processes involved and these are discussed in the subsequent three sections, covering disassembly, cell selection and reconstruction.

#### 2.3.1 Safety standards

System robustness, intrinsically linked to safety, is an important factor. Managing the potential risks stemming from an unregulated SLB market is key to its success, therefore repurposed batteries must meet stringent standards to ensure safe operation. These typically include guidelines for handling, transportation, storage, and operation of SLBs to mitigate risks associated with fire, leakage, and other potential hazards. Regulations and testing standards need to be developed and updated (Christensen et al., 2021) and a variety of global agencies and private-sector coalitions, consisting of Original Equipment Manufacturers (OEMs) and SLB companies, are already working on industry-wide SLB safety standards. These standards would essentially classify batteries based on their performance potential and storage applications based on their performance needs, in order to create transparency into product supply and market demand. Given the dynamic nature of the EV battery industry and the relentless focus on design, manufacturing, and performance breakthroughs, establishing a body to regularly review and refine battery standards and report annually on average cost and operating benchmarks could further catalyse growth in battery deployment.

Certification processes of SLBs ensures compliance with existing regulations. The certification requirements for a SLB system are unclear, particularly as testing standards need to be adapted to cover battery systems built from used battery components, and the varying quality of used batteries affects testing representability (Börner et al., 2022). Standards governing second life should ideally be developed in coherence with those applicable in the first life of batteries, so that companies planning to repurpose batteries perform the same set of tests as for new batteries.

Currently, there no safety standards in the UK or EU specifically for SLBs; however, standards covering the safety assessment of LiBs in applications other than that of their first life (IEC 63330) and high-level guidance on the safe and environmentally sound reuse of LiBs (IEC 63338) are being developed (Christensen et al., 2023). Certain applications, such as the use of second life EV batteries as energy storage for buildings, are currently subject to extensive safety tests. To date, safety testing of SLBs is done under the IEC 62619, which is the reference standard for first life batteries, setting out safety requirements for first life battery design, as well as tests and criteria for evaluating the resilience of batteries to external damage, such as mechanical shock or external short-circuits (IEC 62619-2, 2022). In the United States and Canada, safety evaluation is covered under UL 1974 (UL, 1974, 2018), specifically developed for repurposed batteries.

Regulations requiring data included in the BMS to be openly accessible are needed, as this information is essential to facilitate repurposing of batteries, allowing for greater streamlining of the processes. Key performance indicators, such as the internal resistance, charging/discharging history, occurrence and frequency of high temperature events, among others, enable rapid identification of those packs, modules, and cells retaining sufficient capability to be considered for second life use. Analysis of this data can avoid the need for extensive testing and thus dramatically reduce the costs of repurposing.

Specific performance standards need to be met, in order to select and certify the suitability of repurposed battery cells for their new applications. These standards define parameters such as capacity, efficiency, and cycle life, which determine the battery’s ability to store and release energy effectively.

#### 2.3.2 Safety checks on whole packs

Several processes may occur between the end-of-first-life and beginning-of-second-life, including SoH characterization, disassembly, and remanufacture, as depicted in Figure 1. As disassembly and further processing diminishes the economic benefits of repurposing (see Section 3), there is a strong preference for the full battery pack to be immediately deployed in a second-life application, where possible.

The first step is removal from the EV chassis, so that a post-auto battery assessment can be performed, to assess the suitability of the battery pack for a second-life application. Processes vary from case to case, but typically this assessment would include:

1. Visual inspection for damage;
2. Ensuring functionality in charge and discharge;
3. Complete characterization at battery pack level.

Results from the post-auto battery assessment are used to determine suitability for immediate deployment in a second-life application. If the battery pack passes the post-auto battery assessment, further SoH tests will be conducted to optimise the deployment in a second-life application (see Section 2.3.3). If the battery pack fails the post-auto battery assessment, then further disassembly would occur to assess the health of individual modules within the pack or, in extreme cases, individual cells within each module. The intention here is to recycle as many modules and/or cells from the pack as possible, whilst accepting that some modules and/or cells are degraded beyond any useful second-life application.

Individual modules and/or cells that are still useful for second-life applications would typically be paired with other modules/cells that have been degraded to a similar extent. The processes for this pairing are not covered in this review, although the broad narrative presented below would be applicable at module and cell level.

#### 2.3.3 Evaluating battery pack state-of-health

The second-life battery industry has an established process, whereby all battery packs, once they have passed the post-auto battery assessment, undergo further SoH testing to determine the most suitable second life application. SoH is an ambiguous quantity, not linked to a single measurable, but rather an arbitrary, catch-all state parameter describing the performance of the battery, typically as a percentage of its beginning of life performance. SoH can be as simple as examining the capacity fade–i.e., what capacity (measured in Ampere-hours) of the battery remains accessible, compared to that at beginning of life. On the other hand, SoH may consider power fade, indicated through the increase in battery pack resistance. The United Nations have established the State of Certified Energy (SOCE) metric to define an EV battery’s capability to store energy at a point in its lifecycle, as a ratio of its beginning of life energy storage capacity; this correlates directly to the reduction in driving range. This section introduces processes to define SoH for EV battery packs and discusses the suitability and applicability of each.

The wide range of second-life applications means that the requirements vary enormously. Moreover, each battery will have a unique SoH state, taking into consideration all viable degradation mechanisms, and the range of operational characteristics that it may have been exposed to in its first life. Consequently, the optimisation of battery selection for a given second life application is highly complex. SoH evaluation methods must be able to paint a broad picture of expected performance (through capacity fade and power fade), as well as highlight any potential issues, particularly those associated with safety and thermal runaway.

Simple beginning-of-second-life SoH checks will provide sufficient data for determining the sizing requirements of an SLB battery pack to meet the second life energy and power specifications (Martinez-Laserna et al., 2018; Lam et al., 2022). The capability to examine a battery *ex situ* and in specialised testing centres means that more in-depth SoH evaluation methods become viable, in order to gain the best picture of the battery’s expected future performance (Warner, 2015a).

**FIGURE 2:**
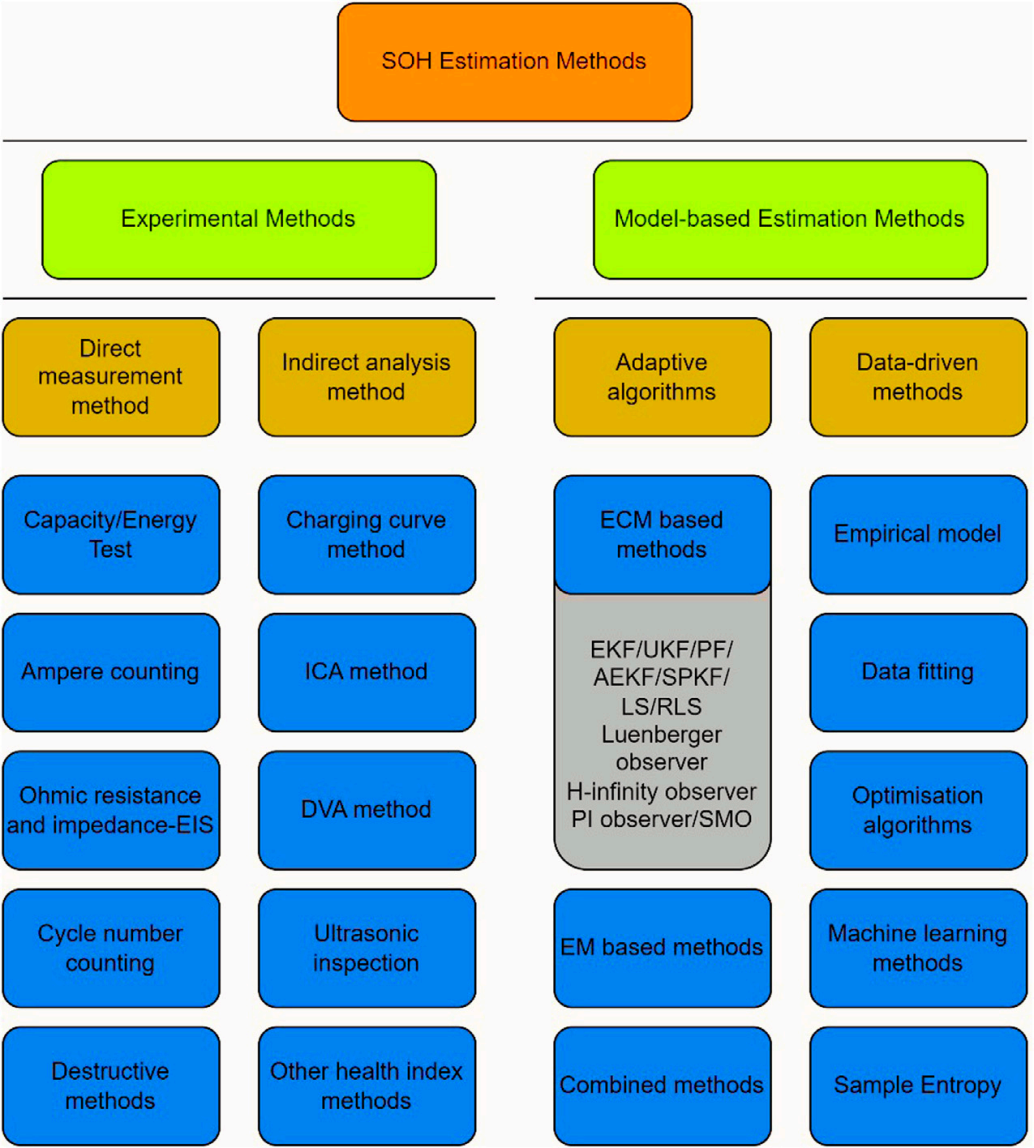
State-of-health (SoH) estimation methods.

Noura et al. (2020) split SoH estimation methods into three groups: Experimental Methods, Model-based Methods and Machine Learning Methods, as summarised in Figure 2. Experimental methods employ empirical methods to extract performance characteristics from the battery. The data may be used directly to indicate SoH (such as accessible capacity), or data processing may extract indirect parameters of the battery, such as resistance. Experimental methods are widely employed and provide an excellent oversight of the battery’s performance. However, they do not offer scope to evaluate far beyond the symptoms of degradation. Modelling methods are predictive, but can be verified through empirical measurements, and have the capability to offer more detail on the expected degradation modes and mechanisms, rather than just the symptoms of degradation (Dechent et al., 2021). Modelling methods may run alongside the battery pack throughout its first life or may be used to assess historical data, usually taken from the BMS. Increasingly, machine learning methods are being employed alongside experimental or modelling methods. Companies such as *Accure GmBH* evaluate enormous volumes of real-world data to observe trends in battery performance, based upon certain operational characteristics (such as the ambient conditions in their country of use, or EV driving style).

Capacity and power fade are indicators of battery health and can be used to estimate future runtime and performance throughout its second life, essential for any stakeholder to choose a repurposed battery over a newly manufactured one. As such, it is essential to be able to predict the further degradation of the battery, i.e., the continued impact on capacity and power fade. Ramoni and Zhang (Ramoni and Zhang, 2013) predict that SLBs (beginning at a capacity equivalent to 70%–80% of its beginning of life capacity) can last 5–10 years in grid-storage applications, although (Zhu et al., 2021a) warn that these numbers rely on being able to detect faults in cells within the battery pack. However, since the most cost-effective way to repurpose “retired” battery packs is to do so with minimal intervention, i.e., without tearing the packs down to cell or even module level, cell-level testing is not usually feasible.

Capacity fade is a consequence of a degraded battery caused by one (or a combination) of three degradation modes: (i) loss of active material (LAM), (ii) stoichiometric drift and/or (iii) loss of lithium inventory (LLI) (Neubauer and Pesaran, 2011a). The simplest and most effective way of determining capacity fade is through Coulomb counting, a procedure consisting of a full, constant current discharge (usually at 1°C and 25°C) to record the amount of charge that passes from the battery and comparing that to its initial rated charge capacity. Coulomb counting, however, does not define the dominant degradation mode, and provides no insight into the degradation mechanism that has led to a reduction in capacity. As a result, used alone, Coulomb counting provides limited insight into the expected future performance of the battery, which is critical for SLB assignment (Montes et al., 2022a).

Incremental Capacity Analysis or Differential Voltage Analysis are SoH diagnostic techniques that predict capacity fade and can identify specific degradation modes and degradation mechanisms by tracking changes in voltage response throughout the battery’s lifetime, for example, lithium plating (Campbell et al., 2019), which can then be used to approximate capacity fade (Wang et al., 2017). Hence, in order for second-life assignment processes to make more thorough evaluations of the risks of future performance failure, and future catastrophic failure, there is a need for the battery’s history to be included in open access battery passports. The vision for a digital battery passport (GBA, 2022) is a log containing a range of information about the battery, such as material composition, performance characteristics and safe disassembly instructions, which is useful to all stakeholders in the battery supply chain. Including the cycling history of the battery during its use would enable effective decision-making for the battery’s end-of-life pathway.

Ren et al. (2019) have shown that a lithium-ion cell’s thermal stability is most compromised when a large amount of lithium plating has occurred (typically associated with cold temperature fast-charging at high state of charge), whilst the thermal stability of lithium-ion cells that have spent their first life at elevated temperatures is actually improved, because of the accelerated growth of the Solid Electrolyte Interphase (SEI) layer (Börner et al., 2017). This further supports the criticality of having historical data which includes thermal conditions, tracked and openly accessible through concepts like a battery passport.

Further SoH understanding can be gained from observing the power fade of the battery. Internal cell impedance increase is the most prominent reason for power fade and is caused by the resistance of the lithium-ion cell increasing during lifetime operation. Resistance increase causes increased overpotential during an excitation of the battery, i.e., whenever charge is passing through the cell. The overpotential can be characterized using techniques such as galvanostatic intermittent titration, where a battery is subjected to constant current pulses and the resulting change in battery voltage is recorded. Through Ohm’s law, the real-time cell resistance can be extracted directly (Kim et al., 2022). Galvanostatic intermittent titration techniques provide good insight of the total battery resistance, as well as measuring the complex impedance, if used alongside model-based methods where the voltage response is fitted to equivalent circuit models (ECM) (LeBel et al., 2022). However, galvanostatic intermittent titration cannot isolate the constituent components of resistance, and therefore cannot relate power fade back to degradation mechanisms.

**FIGURE 3:**
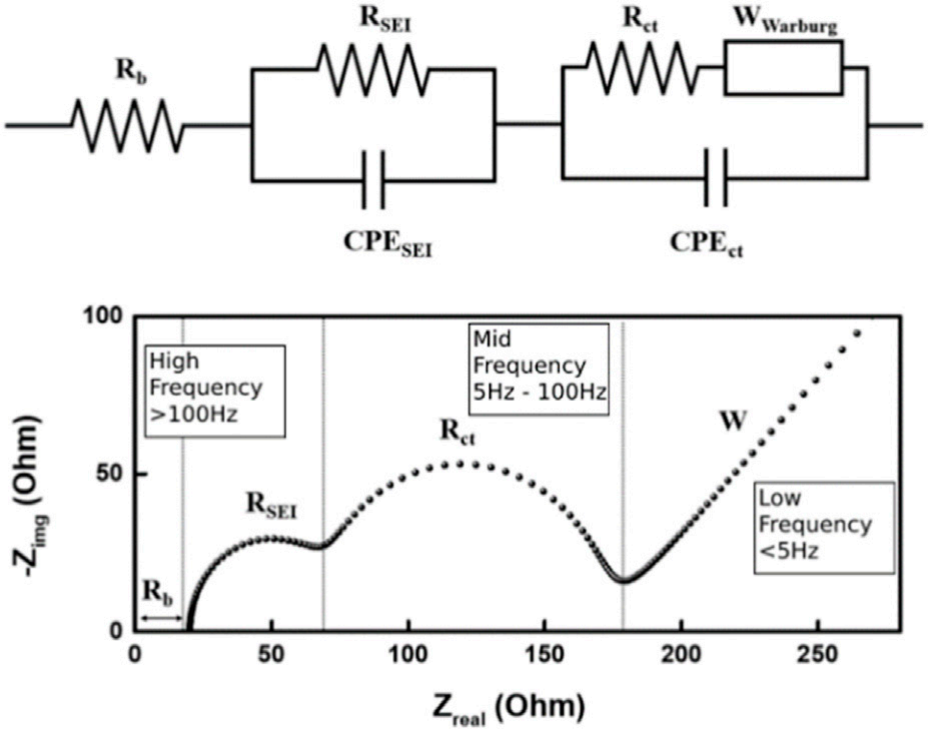
Simple example of an Electrochemical Impedance Spectroscopy plot used alongside an appropriate Equivalent Circuit Model to determine resistance mechanisms and parameters, CPE represents constant phase elements for the Solid Electrolyte Interphase (SEI) and charge transfer mechanisms.

Electrochemical Impedance Spectroscopy (EIS) is able to isolate the individual components of resistance within a cell, thus helping to identify specific degradation mechanisms. EIS consists of passing a small alternating voltage or current at various frequencies through a battery and analyzing the response signal (see Figure 3 for example, of EIS data). This technique requires expensive equipment to allow extremely high precision measurements. Additionally, simultaneous EIS measurements on cells in a battery pack can lead to crosstalk interference with neighboring cells, causing inaccurate results (Raijmakers et al., 2016). Battery packs will need to be dismantled to enable accurate measurements to be carried out at the cell level. This is not a viable option for second-life assessment of batteries.

SoH characterisation is essential for assessing suitability for second-life use and optimisation of how the battery is used by the BMS in the new application. The challenge lies in selecting appropriate methods that are effective in single cell/laboratory experimentation and scaling them to be viable for EV battery testing, so that whole packs can be tested without disassembly. The selection of a suitable SoH characterization method is also dependent on resource (specialist equipment is required for many methods) and, crucially, the expected second life application. It is imperative that research continues into understanding the consequence of certain degradation mechanisms/modes in the performance of LiBs during second-life applications. This will allow the industry to develop characterization methods that focus on those degradation signatures that are most relevant for predicting second-life performance–an important first step in scaling the second-life industry.

Without adequate fault detection at cell level, early onset of pack EoL is unpredictable, as even a single faulty cell can overheat and risk the pack’s integrity, presenting a significant risk to the ongoing operational robustness and safe operation of the battery pack. This places a significant barrier to adoption of SLBs and so predictive diagnostics must play a key part in the future development of viable second life grid storage applications.

### 2.4 Pack degradation grading

A standardized process for grading any EV battery for second-life applications does not yet exist in the UK or EU. However, in the US and Canada, the UL 1974 (Standard for Evaluation for Repurposing Batteries) processes are being developed (UL, 1974, 2018). For example, 4R Energy Corp, a joint venture between Nissan and Sumitomo Corporation and a market leader in second-life battery technology, grades EoL Nissan LEAF batteries into four categories, as listed below.

• **Grade A**—battery shows little signs of degradation and could be reused in a new EV.
• **Grade B**—battery shows some signs of degradation but can still be repurposed for most second-life applications, for example, stationary energy storage.
• **Grade C**—battery is heavily degraded but still functional. They are typically deployed in backup power systems (Hill, 2021).
• **Grade D**—battery is unsuitable for any second-life applications and should be recycled.

The 4R Energy Corp grading process is simplistic and qualitative but does provide a basic framework around which quantitative SoH testing processes should be constructed. There is a wealth of knowledge and understanding of how the SoH of a single cell can be accurately determined; the current challenge for the battery industry is to scale this SoH testing to be widely suitable for full battery packs, so that such a grading system can be effectively informed.

### 2.5 Pack disassembly

When a pack is unsuitable for direct reuse, due to either degradation or safety issues (see Section 2.3.2) or application requirements, it can be refurbished to varying degrees, ranging from replacement of faulty cells to complete disassembly, or combining multiple battery packs to create larger storage systems.

Battery pack disassembly includes the removal of the pack cover, BMS and thermal management components, electronic parts and, finally, the modules and cells. Recently, increasing concerns have been raised regarding efficient battery disassembly processes. In particular, the large variety of pack designs and structural components, such as welded parts and strong structural glues, impede streamlined and fast disassembly (Harper et al., 2019a; Thompson et al., 2020; Lander et al., 2023). Facilitating and accelerating the disassembly process is key to be able to cope with the large amount of EoL batteries expected in the coming decades. Here, alternative design approaches such as clip fasteners or easily separable tapes as well as standardised battery packs could solve these obstacles. Ultimately, the partial or full automation of battery disassembly is an important step towards a time-efficient and cost-effective disassembly process (Hellmuth et al., 2021; Lander et al., 2023).

Battery cells recovered from disassembled packs can be subjected to various operational conditions and stress tests to assess their behaviour and ongoing safety and performance in real-world applications. Thorough processes for cell selection ensures that only EoL battery cells having acceptable energy storage capabilities, minimal degradation, and satisfactory safety characteristics are repurposed. Based on their condition and performance, cells need to be sorted and graded into different categories. This ensures that battery cells with similar characteristics are used together in their second-life application, and that the applications for the battery cells is selected appropriately. First, the battery cells’ fundamental characteristics must be matched–this considers chemistry, voltage and capacity–misalignment of these fundamentals will mean an unbalanced second-life energy storage system (Montes et al., 2022a). Next, battery cells with similar states of degradation must be considered, to ensure the grouped battery cells operate effectively with one another. (Montes et al., 2022a).

Integration with suitable energy management systems, such as a BMS, is essential to monitor and control the performance of repurposed battery packs effectively. Once battery packs are reconstructed, they must undergo rigorous testing and validation to verify their compliance with the safety and performance standards discussed in Section 2.3.1. Comprehensive documentation would be required throughout the repurposing process to demonstrate compliance with any future legal standards that may arise. Detailed records of battery selection, test results, sorting criteria, repurposing procedures, and any modifications made during the process aid in ensuring traceability and compliance.

When repurposing batteries, it is crucial to assess the compatibility of the original battery chemistry with the target application. Different battery chemistries, such as LFP, lithium nickel manganese cobalt oxide (NMC), and lithium nickel cobalt aluminium oxide (NCA), exhibit distinct performance characteristics and varying limitations. These include energy density, power density, cycle life, and thermal stability. Matching the chemistry to the specific requirements of the repurposed application is essential to maximize performance and safety (Warner, 2015b). Finding a chemistry that aligns with the specific requirements of the repurposed application can be challenging. Incompatible chemistry matching may result in suboptimal performance, safety risks, and introduce complexity in battery management.

Voltage and capacity matching with the target application is vital and ensures reliable operation. Incompatible voltage levels or significant variations in capacity can result in inefficient energy transfer and potential safety risks. Aligning the voltage and capacity characteristics ensures seamless integration and reliable operation within the intended system. Repurposing batteries with similar levels of aging and degradation ensures better compatibility in terms of performance and operational characteristics.

Considerable research (in the academic and industrial R&D sectors) is focused on the development of new, sophisticated BMS processes, enabling enhanced diagnostics and monitoring capabilities that will improve the performance and safety of repurposed batteries. Inexhaustibly, such systems in development can detect and manage battery degradation to a greater degree (Offer et al., 2016), monitor load imbalance across the battery packs (Naguib et al., 2021), or identify potential faults before they become safety critical (Zhao et al., 2024). All this will lead towards greater confidence in future generations of BMS, meaning the repurposed battery may be used more expansively, taking greater load, and operated with reduced safety factors before its end-of-life is declared (Montes et al., 2022a). Implementing intelligent energy storage controls that adapt to the characteristics and limitations of repurposed batteries can optimize their operation. These controls can optimize charge/discharge rates, manage thermal considerations, and maximize battery lifespan.

Developing and following a structured approach that includes careful selection, testing, sorting, repurposing processes, testing and validation, documentation, and environmental considerations, SLB projects can successfully meet legal standards and contribute to a more sustainable energy future.

## 3 Economics of second life applications

Energy stored on energy invested (ESOI) is a measure of the returns from a battery’s useful life over the energy spent on manufacturing the battery. It is widely used in industry to measure the effectiveness of an energy storage technology. For EoL batteries used in a second life application, their energy stored on energy invested will be higher than that of a newly manufactured battery.

From an economic point of view, second life competes with battery recycling and the purchase of new batteries and effective business models are crucial for the SLB market to thrive. For second life to be financially viable, the sum of the buying price and refurbishment costs of SLBs would need to be lower than purchasing a new battery.

### 3.1 Second lifes vs. new batteries

The attractiveness of SLBs depends on the price margin between SLBs and new batteries. Only if the price difference is large enough, will deploying a used battery instead of a new one be justified. The market value of SLBs depends on the remaining capacity and overall performance, the battery chemistry, the intended second life application, and the predicted lifetime/defined EoL for second life (Martinez-Laserna et al., 2018; Sun et al., 2018). The longer the SLB lifetime, the higher its value (Martinez-Laserna et al., 2018).

Trading price estimations for SLBs vary across studies and timeframes, from $38/kWh up to $300/kWh (Cready et al., 2003; Neubauer et al., 2012a; Melin, 2019a; Kamath et al., 2020a). Overall, the SLB price is predicted to fall caused by a larger market supply of SLBs and the decreasing cost of new batteries (Engel et al., 2019; Zhao et al., 2021a). It is estimated that, by 2025, SLBs will have a price advantage of 30%–70%, compared to new LiBs (Engel et al., 2019). However, due to the price of new batteries declining faster than for SLBs, this price margin could decrease to 25% (Melin, 2019a; Kamath et al., 2020a). Similarly, Sun et al. (2018) have shown that whilst the price of SLBs is expected to significantly decrease until 2025, the SLB cost will eventually plateau and the price advantage of SLBs compared to new batteries will reduce.

Overall, the buying price of SLBs and the refurbishment costs should be low enough to make profit from selling SLBs below the price of new LiBs. Therefore, if SLB prices or refurbishment costs cannot be further reduced, this option might become less attractive in the future and buying new LiBs might prevail.

### 3.2 Testing and refurbishment costs

**FIGURE 4:**
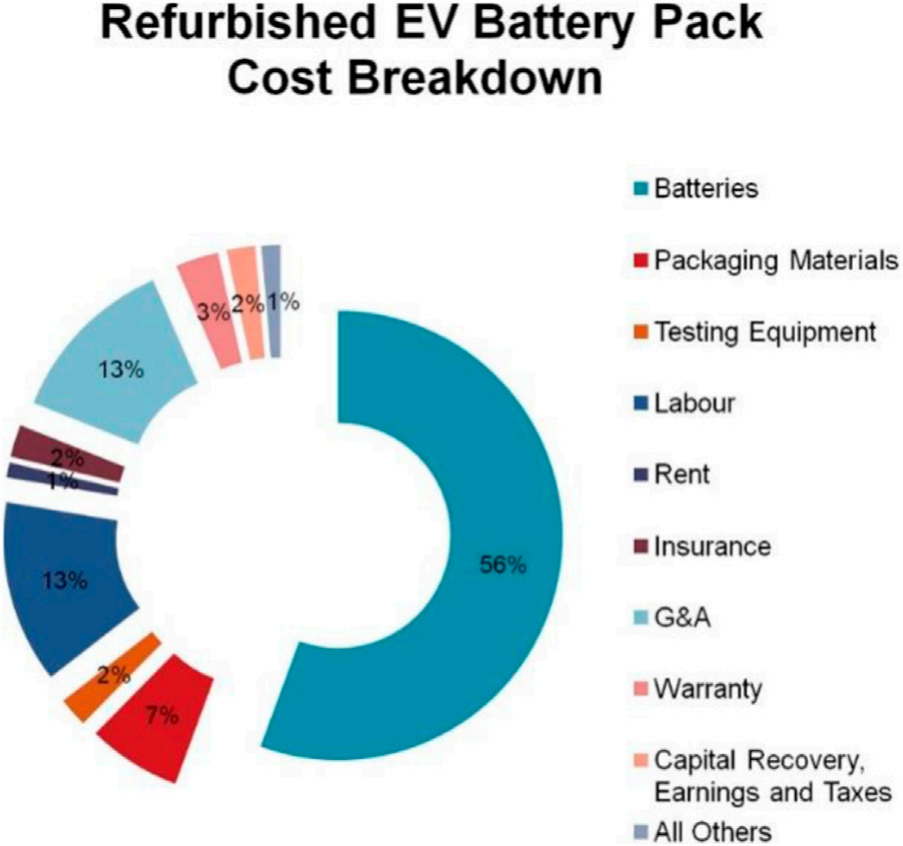
Summary of the costs involved in the refurbishment of EV batteries from the collection to the selling. Reprinted from Martinez-Laserna et al. Renew. Sust. Energ. Rev. 93, 701–718, 2018.

According to available literature on SLBs (Haram et al., 2021a), refurbishment costs range from $12/kWh to $50/kWh (Cready et al., 2003; Neubauer et al., 2012a; Martinez-Laserna et al., 2018). The largest contribution to refurbishment costs comes from buying the battery (Figure 4) (Martinez-Laserna et al., 2018; Zhao et al., 2021a). Furthermore, labour costs make up to 13% of the total refurbishment cost, with the most labour-intense processes being SoH assessment and disassembly. A study by Neubauer et al. (2015) has shown that the incurred labour cost for testing varies significantly with the length of the testing protocol, the handling time, and the size of the battery (in the study, module level was assumed).

**FIGURE 5:**
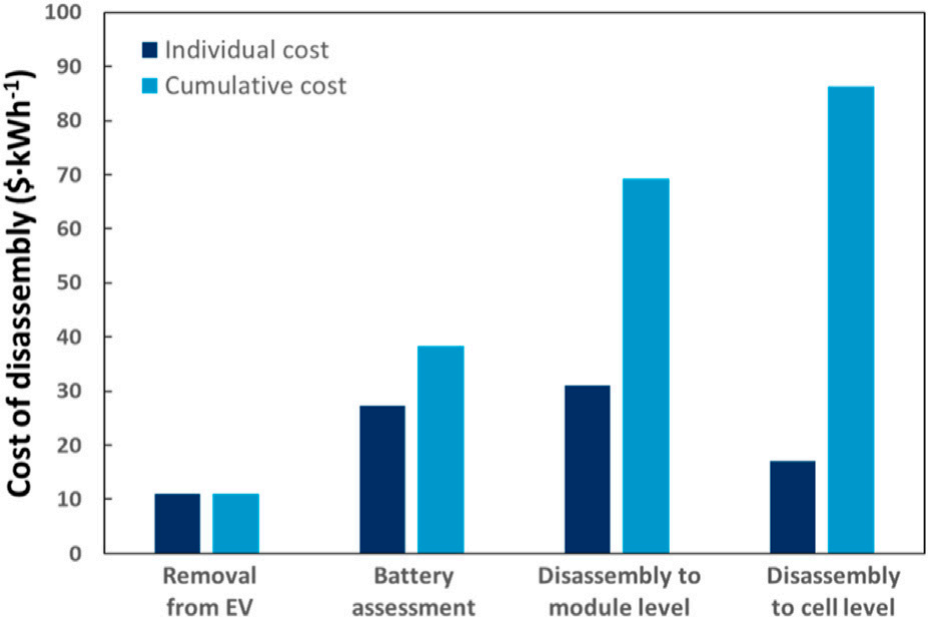
Cost of battery disassembly (in $/kWh) showing the individual cost of each step and the cumulative cost up to the respective disassembly stage. Reprinted with permission from Rallo et al. Resour. Conserv. Recycl. 159, 104785, 2020.

Disassembly costs depend on the intended application and required disassembly level. For instance, in some cases, the battery might be used as is; in other cases, it might be necessary to disassemble the battery down to cell level, or only to module level. Figure 5 highlights that the case for each additional disassembly step becomes increasingly harder to justify (Rallo et al., 2020). The benefit of identifying and replacing the most degraded module and/or cell must be justified against the additional incurred cost and, at a certain point, immediate recycling becomes the better economic decision. A study by Alfaro-Algaba and Ramirez (2020) presents a framework to evaluate the optimal disassembly stage or “disassembly stopping point”, with regards to economic and environmental benefits. Calculations on when the disassembly process reached its financial and environmental peak include the total economic gain minus the disassembly costs and the environmental impact avoided by not disposing of components, minus the environmental impact caused by disassembly processes (Alfaro-Algaba and Ramirez, 2020).

### 3.3 Second life vs. recycling

**FIGURE 6:**
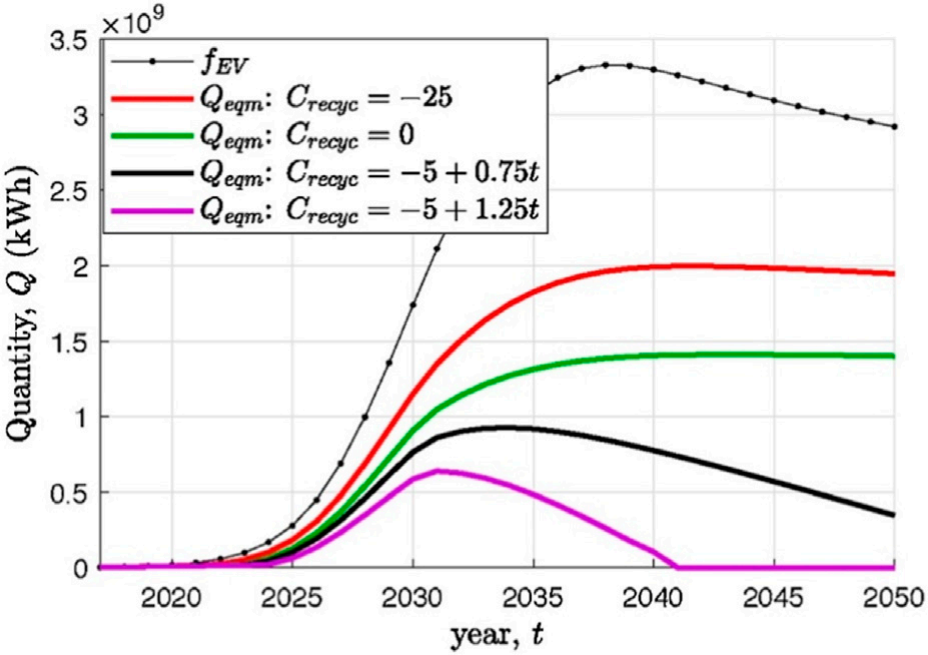
Quantity sold into second-life applications under different recycling net credit scenarios, shown in comparison to the total used EV batteries produced. Reprinted with permission from Sun et al. J. Energy Storage 19, 41–51, 2018.

To economically justify second life applications, the profit made from SLBs should outweigh the recycling profit, otherwise it would be more attractive to immediately recycle EoL batteries. Recycling profits strongly depend on the recycling efficiency, cost of recycling processes, economies of scale, and the value of the recovered materials (Sun et al., 2018; Lander et al., 2023). Especially with new legislations requiring EoL batteries to be recycled as well as for new LiBs to contain a minimum amount of recycled material, an increase in recycling profits is predicted. This, combined with a decreasing cost margin between SLBs and new LiBs, renders the SLB option less attractive. Sun et al. (2018) have shown in their SLB sales simulations that, compared to a predicted recycling profit of $45/kWh in 2050, the profit margin for SLBs with an assumed refurbishment cost of $20/kWh and a sales price of <$50/kWh, is too low and eventually SLB sales will stop (Figure 6).

### 3.4 Impact of second life on the price of new batteries

Reusing retired batteries would increase the salvage value of a battery that otherwise would be at the disposal stage which, in turn, would decrease the upfront cost of such batteries when the EV is purchased (Martinez-Laserna et al., 2018). According to studies by (Neubauer and Pesaran, 2011a; Neubauer et al., 2012a) a discount of 2.2%–25% on the upfront cost would be possible, depending on the assumptions made in the cost model such as initial battery costs, repurposing costs, and the SoH of the battery. However, as battery prices continue to fall annually (Nykvist and Nilsson, 2015), battery costs represent a decreasing share of the total EV upfront costs. Thus, even considering that battery second life use may produce an appealing reduction of the upfront cost, it might be insufficient to change the overall EV costs significantly and to be a disruptive factor in favour of faster EV adoption (Martinez-Laserna et al., 2018). Note that the relationship between EV OEM and the buyer of the SLB should be specified, i.e., is the original seller paid the salvage value or not. Taking into consideration current practices in these models would help to obtain a more realistic scenario of the impact of second life on the EV upfront cost.

### 3.5 Opportunities to increase second life battery profit

To maintain profitability for the SLB pathway, various strategies can be followed: i) SoH assessment has been shown to be highly labour- and thus cost-intensive. Avoiding these costs by using battery passports might be a potential way of reducing refurbishment costs. ii) Further, costs associated with collection and transportation of EoL batteries or SLBs can be reduced by keeping logistics local, i.e., setting up a refurbishment plant close to an EoL collection site (Neubauer et al., 2015). iii) Labour cost is a significant cost factor in the refurbishment phase. Here, SLBs might be lucrative either using automated refurbishment processes (Zhao et al., 2021a; Zhu et al., 2021a). Generally, easier to disassemble battery packs would further reduce the refurbishment costs.

## 4 Environmental considerations for second life applications

There are environmental benefits to repurposing batteries, but there may also be drawbacks. Increasing the service life of LiBs reduces the overall life cycle environmental impact from battery manufacturing (Jiao and Evans, 2016a; Harper et al., 2019a), and second life use displaces the impacts from manufacturing a new battery of similar capacity (Haram et al., 2021a). However, there is an argument for recycling batteries containing cobalt immediately, without extending their already long lives further through second life use (Harper et al., 2019a). This would serve to recover cobalt faster, displacing the impacts through extraction of their ores and, since cobalt is a valuable material, enabling profitability for the developing recycling industry. This argument could also apply to other high impact materials, such as nickel, the conflict between these two options is still being debated (Gaines, 2018; Harper et al., 2019a; Dunn et al., 2021).

**FIGURE 7:**
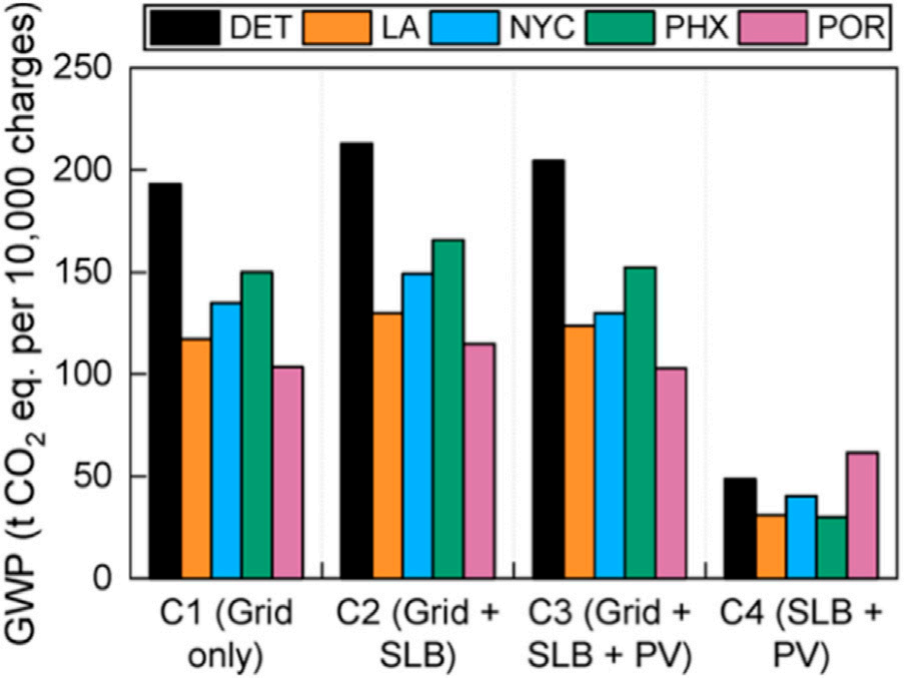
Global warming potential (GWP) for each configuration at 100 kW charging power for five U.S. Cities [DET, Detroit; LA, Los Angeles; NYC, New York City; PHX, Phoenix; POR, Portland. Adapted from Kamath et al. Environ. Sci. Technol. 54, 6878–6887, 2020.

The processes of disassembly and remanufacture for second life use also add environmental burdens, although these are considered to be much smaller than those for manufacturing new batteries (Cicconi et al., 2012b). Several studies have analysed the environmental benefits of SLBs. (Haram et al., 2021a) lists excessive use of raw materials, water and electricity which contribute to CO_2_ emissions. (Dunn et al., 2023) found repurposing and reuse to be favoured overall, but they do not account for the impacts of the delayed recycling opportunity. The study by (Kamath et al., 2020a) compared EV fast-charging using power from the grid to that from BESS using second life LiBs, in terms of economic cost and life cycle environmental impact in five U.S. cities. The results are presented in Figure 7 below. Compared to using new batteries, they found that SLB reduced the global warming potential (GWP) by up to 77% (Kamath et al., 2020a).

Kamath et al. (2020b) also showed that, compared to using new batteries, combining SLBs with renewable energy sources can further reduce the GWP of the fast-charging system by reducing the grid electricity use, the reduction amount depending on the location and the charging power of the system. Casals et al. (2015) described how battery usage is only advisable in association with renewable energy sources, with a reduction of 32% GWP when EV batteries are reused in island installation, compared with using new lead-acid batteries. Cicconi et al. (2012b) considered the environmental performance of an EV battery when repurposed in a smart grid providing peak management, power quality and reliability services, comparing this to an EV battery which is disposed of after automotive use. They assumed that disassembly and remanufacturing impacts are negligible and accounted for the reduced efficiency of SLBs, as well as replacements needed to ensure that the SLB services the whole application lifespan, finding that second life use resulted in a 25% reduction in GWP (Cicconi et al., 2012b). Ioakimidis et al. (2019) performed a similar study for the use of an LFP EV battery for supplying electricity to buildings, finding a potential 15% reduction in GWP when the EV battery is used to store energy from solar panels, compared with manufacture of a new LFP battery. A study by Richa et al. (2017) showed that the benefits of repurposing EV batteries for another 5–7 years, compared to manufacturing and installing new lead-acid batteries, could reduce the gross energy demand and GWP by 15%–70% (Richa et al., 2017). However, authors did not consider battery collection, refurbishment, and transportation, which would increase the environmental impacts (Martinez-Laserna et al., 2018). Retrieval after second life could be particularly difficult, if that second life is in a developing country which has no recycling infrastructure.

## 5 Policy

The rising global uptake of electric mobility is undoubtedly beneficial for environmental and climate change goals, but it is equally crucial to be aware of, and mitigate, the associated costs of this transition. Effective governance and regulatory mechanisms to ensure appropriate end-of-life management of batteries, which are among the costliest components of EVs and a rich storehouse of many critical technology metals, will be necessary.

A central aim of product regulation is to maximise safety and minimise human and environmental harm. Although reuse offers promising opportunities for maximising battery value and revenues, widespread EV battery repurposing also carries potential safety risks which need to be mitigated by regulation. Equally important is proper stewardship and recovery of valuable battery materials at the end of life, given the rising threats to future supplies of critical minerals for ongoing manufacture. This section briefly outlines the current UK regulatory framework for EV batteries and discusses the need for more comprehensive and considered regulation to address the challenges of battery second life.

### 5.1 UK battery regulations: present landscape

The primary EU regulations for batteries and end-of-life EV batteries were, until August 2023, contained in the Batteries Directive 2006 (2006/66/EC) (European Parliament, 2006b) and the End-of-Life Vehicles (ELV) Directive (2000/53/EC) (European Parliament, 2000). Although the EU has, in August 2023, repealed the 2006 Batteries Directive and replaced it with the new Batteries Regulation (European Parliament, 2023), the UK has yet to change or update its battery regulatory frameworks (at the time of writing). As new EU regulations no longer automatically apply in a post-Brexit UK, the Waste Batteries and Accumulator Regulations 2009 (SI 2009/890), which transposed the 2006 EU Directive into UK national law, still remains in force in the United Kingdom.

The 2006 EU Batteries Directive and the UK’s 2009 Waste Batteries and Accumulator Regulation were drafted well before the rise of EV and LiB technologies, leaving unaddressed a number of particular challenges raised by these technologies, which was one of the concerns that prompted the EU to replace the 2006 Directive with the 2023 Batteries Regulation (European Parliament, 2023). The new EU Batteries Regulation (Regulation 2023/1542), which emphasises measures for sustainable battery value chains, were first published as proposals in 2020 (European Parliament, 2020), provisionally agreed in January 2023, approved by the European Parliament on 14 June 2023 (European Parliament Press Release, 2023) and came into force in August 2023.

The Regulations introduce, amongst a raft of other changes, a separate distinct category for EV batteries (under the previous Batteries Directive 2006, EV batteries fell under the category of “industrial batteries” rather than automotive batteries due to definitional oddities). The Regulations also set requirements for critical materials recovery, access to BMS data and the introduction of battery passports from 2027. A battery passport, or digital representation of the battery, will convey information about its history, ESG and lifecycle requirements, thus improving transparency and information-sharing across the battery value chain. It is hoped that these changes will facilitate sustainable end-of-life management, as well as help make sounder decisions about battery use, reuse and safety testing. Although the new regulations do introduce some regulatory safeguards for second use (which were missing altogether from the previous Directive), it is questionable whether these go far enough to comprehensively address significant risks from battery repurposing.

The 2023 EU batteries regulations have, as mentioned earlier, no applicability in a post-Brexit UK, which will remain governed by the 2009 legislation until this is replaced by new UK-specific legislation. However, it is doubtful that the UK automotive industry will want to deviate noticeably from EU batteries regulations, given that the EU is one of its largest markets. The UK government’s Department for Environment, Food and Rural Affairs (Defra), is, at the time of writing, carrying out consultations and has commissioned research to revise UK batteries regulation, but there is no clarity yet about when this process will be completed (Grobler, 2022). A number of regulatory gaps continue to surround LiB repurposing operations in the United Kingdom. For reasons outlined below, is crucial that UK regulators act swiftly to ensure proper governance frameworks are in place before large volumes of EV batteries “retire” and are deployed into unregulated second use or repurposing.

### 5.2 Regulating to ensure safer repurposing

Some analysts describe BESS as one of the most important emerging risks today (Hesler and Travers, 2019), and the potential for fire and explosions have already been outlined previously in this article. Worryingly, there is at present little clarity about specific standards for LiB design at first use in the United Kingdom, let alone for repurposed LiBs.

The 2023 EU Battery Regulations aim to address this gap by introducing a number of safety stipulations around safety testing and reporting. Of particular ongoing concern for LiB repurposing, however, is the inadequacy of regulation or standards around domestic LiB installations for energy storage (this issue still remains unaddressed in the new EU Regulations). The significant risk to domestic premises and human life demands strong safeguards, so it is unfortunate that the new regulations make no specific reference to domestic installation and maintenance (Ahuja et al., 2021a). While such specific safeguards may have been seen as unnecessary before the rise of electric mobility, because owners of a BESS would earlier have usually been companies with specialist expertise, these repurposed batteries can now be bought by property developers, councils or other parties with limited understanding of LiB hazards or how to manage them. This regulatory gap could, in the future, have potentially disastrous consequences for residents, neighbours, and bystanders (Ahuja et al., 2021a). Regulations to ensure safety testing for battery repurposing, as well as guidelines for safe installation and maintenance of repurposed batteries, are a crucial prerequisite to such operations in the United Kingdom.

### 5.3 A sustainable value chain for repurposed lithium-ion batteries

Another major question that remains unanswered in the new EU Regulations is about who owns and bears responsibility for batteries that go into second use: a crucial question from the standpoint of battery circular economy as well as legal liability.

Electric vehicles typically use six times more critical minerals than traditional automotives, and other green technologies such as wind turbines similarly require large quantities of these materials. Therefore, the global shift to electric mobility and green technologies is predicted to lead to a massive surge in critical minerals demand (Kim, 2021). Recognising the significance of this issue, the UK Government in 2022 published its first ever Critical Minerals Strategy (HM Government, 2022a) which emphasises the need to bolster domestic production of technology minerals through both increased mining and circular economy strategies for critical materials recovery. This will be supported by the newly established UK Critical Minerals Intelligence Centre (UKCMIC) (HM Government, 2022b) which will analyse stocks and flows of critical minerals. It is crucially important, therefore, that second life operations are carefully steered to ensure recovery of battery minerals.

Materials stewardship for vehicles and components has so far been enshrined in UK batteries regulation through the 2009 Waste Batteries Regulations, wherein producers are required to meet battery take-back and recycling targets. Additionally, the ELV Directive obliges vehicle manufacturers to take back 85% (by weight) of the products they place on the market to reuse, recycle or remanufacture (European Parliament, 2006a). These regulations impose principles of Extended Producer Responsibility (EPR) on vehicle manufacturers. EPR is a regulatory tool by which manufacturers of certain polluting products are required to also take responsibility for their end-of-life management. Interestingly, reuse counts towards the fulfilment of EPR targets, which suggests that producer obligations are discharged at the point at which the EV battery is repurposed, but this raises a difficult question: who presently bears the responsibility for that battery when it leaves that second life or repurposed use (Dawson et al., 2021)? The ELV Directive regulations only applies to vehicles, so if an EV battery is put into a different form of second use or into a different product via repurposing (e.g., energy storage), it would be difficult to argue that these regulations would still apply. If UK regulation remains unclear on questions of ownership and responsibility for repurposed batteries, this opens the risk of significant numbers of batteries getting “lost” or disappearing at the end of second life and consequently never making it back to the recycling or materials recovery phase (Ahuja et al., 2021a). Similar uncertainties surround questions about liabilities flowing back to the vehicle or battery manufacturers, were repurposed batteries to fail or cause injury or damage (Elkind, 2014).

A further important question (at least in the short-term) is whether LiB repurposing facilitates or hinders sustainability goals. It is generally accepted that reuse ranks as superior to recycling in traditional waste management principles, as illustrated by the EU waste management hierarchy. (European Commission, 2005). Undoubtedly, LiB repurposing offers opportunities to maximise battery lifetime and value, and thus to improve the economics of EoL. However, given the worries around critical mineral supplies for LiB manufacture, it is also important to remain mindful that locking up LiBs in several additional years of second use will inevitably delay their recycling. This, in turn, would delay the recirculation of valuable metals (Tao et al., 2021a), whose supply chains can become more vulnerable to disruption in the near future. The World Economic Forum of 2023 has raised concerns about future global economic warfare over battery minerals in the coming decade (World Economic Forum, 2023). Although the new EU regulations (while acknowledging recycling trade-offs) seem keen to support repurposing, further research is imperative. Future policy around repurposing must be informed by life cycle analysis and economic modelling; regulatory frameworks for repurposing must focus on using critical resources to best advantage (Ahuja et al., 2021a).

There is a danger that many of the environmental gains from the transition to EVs will be lost if the UK fails to manage EV LiBs effectively at the end of their useful life. An Insight report by the Faraday Institution (Lee and Gifford, 2020) recommends a high level regulatory and policy framework for LiB reuse to include clear regulation for safety and a battery circular economy, including the influencing of business ownership models (e.g., battery leasing schemes (Ahuja et al., 2020)) to facilitate recycling and second use.

Clear regulations and standards are a prerequisite to widespread EV LiB second use. Sound regulation will need to balance safety and sustainability considerations while negotiating trade-offs between wider economic and environmental constraints.

## 6 Current outlook and challenges

The current outlook for second-life batteries is promising, with increasing interest and research focused on their repurposing potential. As the demand for energy storage solutions continues to grow, repurposing batteries offers a cost-effective and sustainable option. Improving and accelerating progress in second life pathways will lead to increased efficiency, reduced environmental impact, and wider adoption of sustainable energy storage solutions. However, there are crucial challenges to overcome with engineering, economics and policy, requiring greater collaboration among researchers, battery manufacturers, and stakeholders to develop industry-wide standards and best practices for battery repurposing, promoting consistency, interoperability, and safety across different sectors and applications. Several areas, listed below, can be improved to enhance the utilization of second-life batteries.

### 6.1 End-of-life determination

The determination of the EoL for a LiB based on the current SoH criteria is not universally agreed upon, and opinions vary regarding the specific threshold. The 80% capacity fade criterion is commonly used in the automotive sector today, but there is ongoing debate on whether it should be re-evaluated, particularly as rapid advancements in LiB technology have led to batteries with improved degradation characteristics and longer lifespans. These advancements may render the 80% SoH criterion outdated or overly conservative for certain battery chemistries and, since different applications tolerate lower SoH values without significant compromises on performance or safety, reviewing this threshold is imperative to maximising the full potential of a battery.

### 6.2 State-of-health diagnostics

Advancements in battery testing and characterization techniques can improve the assessment of battery SoH and performance, enabling better selection and matching of repurposed batteries with suitable applications. This includes the development of non-destructive testing methods and standardized protocols for evaluating the remaining capacity, internal resistance, and other relevant parameters of SLBs. A particular focus should be placed on the suitability of health diagnostic methods at battery pack level, because the cost associated with dismantling a battery pack to run cell-level diagnostic methods renders many 2nd life applications non-viable. In particular, access to the data stored within the battery management system and through battery passports will facilitate end-of-life decision-making, repurposing and recycling processes more cost-effective, safer and more sustainable.

### 6.3 Battery design

There is a need for battery packs to be designed with EoL requirements in mind, referred to as eco-design or Design for Disassembly, which will simplify refurbishment, repurposing and recycling processes. The use of reversible fastening mechanisms will reduce disassembly times and modular battery pack configurations will allow for easier replacement of individual cells or modules, extending the overall lifespan of the battery system. The development of standardized battery interfaces and communication protocols will enable seamless integration of repurposed batteries into various battery storage systems, regardless of the original battery manufacturer or chemistry.

### 6.4 Circular economy

Establishing a robust battery recycling infrastructure will promote the recovery and reuse of valuable materials from EoL batteries and reduce the reliance on raw materials, minimizing the environmental impact of battery manufacturing. Conducting comprehensive life cycle assessments of repurposed batteries helps quantify their environmental benefits and identify areas for further improvement. This assessment includes factors such as energy consumption, emissions, and waste generation throughout the battery’s entire lifespan.

The economics of SLBs are a balancing act between the margin comparable to new LiBs, additional costs for refurbishment, and the competition with recycling. For SLBs to be fully economically viable, the refurbishment and logistics (transportation, collection) costs would need to decrease. However, the full costs depend strongly on future technology, policy and market developments, and are difficult to predict at such early stages. A more comprehensive assessment is needed to assess whether the environmental and economic benefits of repurposing outweigh the benefits of extending the battery’s first life (either by decreasing the EoL threshold from 80% or improving the battery technology), or directing EoL batteries immediately to recycling to recover valuable materials.

### 6.5 Policy support for battery repurposing

Policy frameworks that promote safe repurposing of LiBs can facilitate sustainable energy storage solutions. Governments can offer incentives, funding, or regulatory support to facilitate the repurposing of batteries, promote recycling practices, and ensure stewardship of critical materials. It is crucial that a robust regulatory framework for LiB repurposing prioritises safety and a sustainable battery value chain. Establishing clear regulations and standards for the repurposing process will improve safety and reduce environmental threats. Guidelines for battery selection, testing, and integration, as well as proper recycling and disposal practices will facilitate broader adoption and replication of successful repurposing practices. Equally important are measures to ensure that repurposed batteries are collected and eventually returned to recycling or further reuse at the end of second life. Repurposing an EV battery for use in a non-EV application could mean that they are no longer subject to End-of-life Vehicle regulations for collection and recycling, thus regulatory clarity about responsibilities and obligations at the end of second life would enhance environmental and economic value.
