# Corrigendum: Lithium-ion battery second life: pathways, challenges and outlook

**DOI:** 10.3389/fchem.2024.1414996

**Published:** 2024-04-24

**Authors:** Anisha N. Patel, Laura Lander, Jyoti Ahuja, James Bulman, James K. H. Lum, Julian O. D. Pople, Alastair Hales, Yatish Patel, Jacqueline S. Edge

**Affiliations:** ^1^ Department of Mechanical Engineering, Imperial College London, London, United Kingdom; ^2^ Department of Engineering, King’s College London, London, United Kingdom; ^3^ Birmingham Law School, University of Birmingham, Birmingham, United Kingdom; ^4^ Department of Mechanical Engineering, University of Bristol, Bristol, United Kingdom; ^5^ Department of Engineering, University of Exeter, Exeter, United Kingdom; ^6^ The Faraday Institution, Didcot, United Kingdom

**Keywords:** lithium-ion battery, end-of-life, second life, repurposing, state-of-health, safety, policy, regulation

In the published article, there was an error in the author list. Anisha N. Patel was erroneously excluded as shared senior author. The correct author list appears above. (^†^These authors share senior authorship.)

In the published article, there was an error in the **Funding** statement. The funding for open-access publication of the article had not been confirmed and therefore not been included. The correct statement appears below:

“The authors declare financial support was received for the research, authorship, and/or publication of this article. Funding sources supporting this work came from University of Bristol Postgraduate Research Scholarships to JB and Faraday Institution (grant number FIRG059) to JE and AH. Open access of this publication was funded by the Imperial College London Open Access Fund.”

The authors apologize for these errors and state that this does not change the scientific conclusions of the article in any way. The original article has been updated.

